# Serum Cholesterol Efflux Capacity in Age-Related Macular Degeneration and Polypoidal Choroidal Vasculopathy

**DOI:** 10.1016/j.xops.2022.100142

**Published:** 2022-03-16

**Authors:** Yasuo Yanagi, Richard M.C. Yu, Waseem Ahamed, Marco Yu, Kelvin Yi Chong Teo, Anna C.S. Tan, Ching-Yu Cheng, Tien Yin Wong, Rajendra S. Apte, Chui Ming Gemmy Cheung

**Affiliations:** 1 Singapore National Eye Centre, Singapore Eye Research Institute, Singapore, Republic of Singapore; 2 Academic Clinical Program, Duke-NUS Medical School, National University of Singapore, Singapore, Republic of Singapore; 3 Department of Ophthalmology, Washington University School of Medicine, St. Louis, Missouri; 4 Department of Medicine, Washington University School of Medicine, St. Louis, Missouri; 5 Department of Developmental Biology, Washington University School of Medicine, St. Louis, Missouri

**Keywords:** Age-related macular degeneration, Cholesterol efflux, Drusen, Lipoprotein, Polypoidal choroidal vasculopathy, AMD, age-related macular degeneration, eAMD, early age-related macular degeneration, HDL, high-density lipoprotein, LDL, low-density lipoprotein, nAMD, neovascular age-related macular degeneration, NMR, nuclear magnetic resonance, PCV, polypoidal choroidal vasculopathy, RPE, retinal pigment epithelium, RPMI, Roswell Park Memorial Institute, SCES, Singapore Chinese Eye Study, SD, standard deviation, tAMD, typical neovascular age-related macular degeneration, VLDL, very low-density lipoprotein

## Abstract

**Purpose:**

To investigate serum cholesterol efflux capacity (the ability of the serum to accept cholesterol) and factors that regulate it using nuclear magnetic resonance-quantified measures of lipoprotein particle composition and size and apolipoproteins metrics in patients with age-related macular degeneration (AMD).

**Design:**

Case-control study.

**Participants:**

Four hundred two serum samples from 80 patients with early AMD (eAMD), and 212 patients with neovascular AMD (nAMD), including 80 with typical nAMD (tAMD) and 132 with polypoidal choroidal vasculopathy (PCV), and 110 age- and gender matched control participants.

**Methods:**

Serum from participants showed cholesterol efflux capacity measured using in vitro cell assays and lipoprotein subfractions measured using nuclear magnetic resonance (Nightingale, Ltd). Associations between cholesterol efflux capacity (measured in percentage) and lipid subfractions were investigated in the patients and control participants.

**Main Outcome Measures:**

Cholesterol efflux capacity and lipid subfractions in control, eAMD, and nAMD. Associations between HDL subfractions and cholesterol efflux capacity.

**Results:**

Cholesterol efflux capacity was higher in patients with eAMD (68.0 ± 11.3% [mean ± standard deviation]) and nAMD (75.9 ± 27.7%) than in the control participants (56.9 ± 16.7%) after adjusting for age, gender, and use of lipid-lowering drug (*P* < 0.0001). Nuclear magnetic resonance lipidomics demonstrated that the mean diameter of HDL was larger both in eAMD (9.96 ± 0.27 mm [mean ± standard deviation]) and PCV (9.97 ± 0.23 mm) compared with that of the control participants (9.84 ± 0.24 mm; *P* = 0.0001 for both). Among the 28 HDL subfractions, most of the small, medium, and large HDLs, but none of the 7 extra large HDLs fractions, were associated moderately with cholesterol efflux capacity in eAMD and PCV (*R* = 0.149–0.277).

**Conclusions:**

Serum cholesterol efflux capacity was increased in eAMD and PCV, but not tAMD, possibly reflecting differential underlying pathophysiologic features of lipid dysregulation in tAMD and PCV. Further studies should be directed toward investigating the diverse biological activities of HDL in AMD, including macular pigment transport, regulation of inflammation, and local cholesterol transport system.

Age-related macular degeneration (AMD) and cardiovascular disease have long been hypothesized to share common pathophysiologic features (i.e., the common soil theory).^1^, ^2^, ^3^ In this regard, since the discovery of the association between genetic polymorphism of lipid and lipoprotein metabolism genes and AMD,^4^, ^5^, ^6^ lipid metabolism had been recognized as an important pathway in the pathogenesis of AMD.^7^^,^^8^ However, epidemiologic studies demonstrated that elevated levels of plasma high-density lipoproteins (HDLs), among other lipoproteins, are associated with an increased risk of early and advanced AMD.^9^^,^^10^ Mendelian randomization analysis confirm that high HDL is related causally to AMD.^11^^,^^12^ This paradoxical relationship, in which high HDL levels may be associated with AMD, but are protective of cardiovascular disease, remains an issue of contention and is a focus of emerging research.

Recent studies indicate that the functional properties of HDL, rather than the levels per se, better predict cardiovascular risk.^13^, ^14^, ^15^ Indeed, the composition and particle’s structure, particularly HDL size, as measured by various methods, including nuclear magnetic resonance (NMR),^16^ are more critical for its function than the cholesterol content of HDL.^17^^,^^18^ HDL, which comprises phospholipids, cholesterol, and proteins, prototypically promotes cholesterol efflux by accepting the cholesterol released from the cells and reduces excessive cellular cholesterol content.^19^ Therefore, cholesterol efflux is an antiatherogenic mechanism that has been associated with various metabolic diseases, including cardiovascular diseases. Now, it is generally accepted that small HDL particles promote cholesterol efflux efficiently.^20^ Although, in theory, an increase in HDL cholesterol levels should translate into a reduction in cardiovascular events, recent large clinical trials testing drugs that raise HDL cholesterol levels have not shown a convincing impact on reducing cardiovascular risks.^21^ Hence, a need exists to study the cholesterol homeostasis further in both the context of cardiovascular diseases and also AMD.^22^, ^23^, ^24^ Additionally, HDL has several other functions. For instance, multiple complement-regulatory proteins and a diverse array of distinct serpins have been identified within HDL, and such components may have roles in regulating the complement system and protecting tissue from proteolysis.^25^ HDL also has diverse functions in the pathogenesis of AMD. For example, selective capture of macular pigment carotenoids and its delivery to Müller cells, the main reservoir of these pigments, is related to lipoproteins.^26^, ^27^, ^28^ Such diverse functions by HDL are beginning to be elucidated, yet in the context of AMD, even the most basic aspect of HDL function, that is, cholesterol efflux capacity, has not been explored directly.

It is possible that serum cholesterol efflux capacities in AMD may be different from those of healthy control participants. First, serum HDL was associated with an increased risk of AMD and drusen load in the European Eye Epidemiology Consortium study.^29^ Additionally, high-dose statin resulted in regression of drusen in a high-risk subgroup of patients with AMD.^30^ A double-masked randomized controlled study using high-dose lipophilic statin (simvastatin 40 mg/day) also demonstrated a significant decrease in the risk of progression to advanced AMD or in severity of nonadvanced AMD in the simvastatin group^31^; however, neither pooled results from major epidemiologic studies nor the Age-Related Eye Disease Study 2 reported a statistically significant association between statin use and progression to late AMD.^32^^,^^33^ These seemingly conflicting results may indicate that a specific statin regimen may lower the risk of progression to neovascular AMD (nAMD). Furthermore, AMD is a disease associated with low-grade chronic inflammation, and intravitreal anti-VEGF treatment reportedly influences such systemic factors.^34^ Because inflammation leads to multiple changes in the HDL structure and function, whether the serum cholesterol efflux capacity in patients with AMD changes even after anti–vascular endothelial growth factor treatment merits further investigation. We hypothesized that differences in serum cholesterol efflux capacity exist between healthy control participants and patients with AMD.

HDL, among other lipoprotein particles, may function to promote unesterified cholesterol release from the retinal pigment epithelium (RPE). As such, we investigated cholesterol efflux capacity in AMD using a case-control study design, comparing serum samples from patients with early AMD (eAMD) and nAMD (including 2 subtypes of nAMD: typical nAMD [tAMD] and polypoidal choroidal vasculopathy [PCV]) with age-matched control participants. Because tAMD eyes are believed to have higher levels of drusen load compared with PCV eyes, we investigated whether differences exist in the pattern of relationship between cholesterol efflux and tAMD compared with PCV.^35^ Finally, we further investigated the factors that regulate it using NMR-quantified measures of lipoprotein particle composition and size and apolipoproteins metrics in patients with AMD.

## Methods

### Study Design and Approval

We conducted a case-control study of stored serum samples using patients with nAMD (tAMD and PCV) collected through the Asian AMD Phenotyping Study^36^ and patients with eAMD and age-matched control participants from the Singapore Chinese Eye Study (SCES), a population-based survey of major eye diseases in Singaporean Chinese people.^37^ The studies followed the tenets of the Declaration of Helsinki. Written informed consent was obtained from all participants. Institutional review board approval was obtained from the institutional review board of SingHealth and Singapore Eye Research Institute. Blood collection was performed at the baseline visit in all participants.

### Clinical Evaluation

All patients with nAMD underwent funduscopic examination and OCT as described previously. Fluorescein angiography and indocyanine green angiography (Heidelberg Spectralis HRA; Heidelberg Engineering) was used to confirm the diagnosis of tAMD and to differentiate PCV, as previously described.^36^

#### Patients with Neovascular Age-Related Macular Degeneration (Typical Neovascular Age-Related Macular Degeneration and Polypoidal Choroidal Vasculopathy)

Each patient was examined at baseline as described previously.^36^ The examination procedures included comprehensive ocular examinations (visual acuity, dilated fundus examination, color fundus photography, fluorescein angiography, indocyanine green angiography, and spectral-domain OCT). Polypoidal choroidal vasculopathy and tAMD were diagnosed clinically and confirmed by ocular imaging results, which were graded by trained retinal specialists (Y.Y., K.T., A.T., T.Y.W., and G.C.). Typical nAMD was characterized by exudative changes resulting from the presence of macular neovascularization as confirmed by fluorescein angiography and indocyanine green angiography. Diagnosis of PCV was based on criteria by the EVEREST study group^38^, which comprise the presence of focal subretinal hyperfluorescence on confocal indocyanine green angiography within the first 6 minutes, plus 1 of the following criteria: nodular appearance of polyp(s) on stereoscopic examination, hypofluorescent halo around nodule(s), presence of a branching vascular network, pulsation of polyp(s) on dynamic indocyanine green angiography, orange subretinal nodules on color fundus photography, or massive submacular hemorrhage.^38^

#### Patients with Early Age-Related Macular Degeneration and Control Participants

All participants underwent an interview, systemic examination, and laboratory investigations to determine socioeconomic, ocular, and systemic risk factors according to a standardized protocol derived in part from the SCES.^37^ We used a digital fundus camera to capture color photographs of each eye after pupil dilation. Adequate quality of a fundus photograph for the assessment of AMD status was available for 1 eye at least in each of 3312 participants (98.8%) from the SCES cohort. The Centre for Vision Research, University of Sydney, performed AMD grading using a modification of the Wisconsin Age-Related Maculopathy Grading System,^39^ which defines eAMD as either soft indistinct or reticular drusen or both soft, distinct drusen, plus RPE abnormalities. Patients with eAMD and age- and gender-matched individuals free of any stage of AMD were selected as control participants for this study. The grading was performed by multiple readers who had detailed instructions and dedicated training.

### Sample Size Calculation

Because no previous data were available to use to estimate the number of samples needed for the current study, we initially used serum from 132 treatment-naïve patients with PCV from an AMD phenotyping study and 110 age- and gender-matched control participants from the SECS to compare the serum cholesterol efflux capacity between patients with PCV and control participants without sample size calculation. Based on the results from the initial analysis, sample size calculation was carried out for eAMD and nAMD, including tAMD and PCV. Minimal sample size to detect differences with a statistical power of 90% (α = 0.05) with a 2-tailed test required 80 each of patients with eAMD and nAMD. Regarding nAMD, we decided to perform additional testing on 80 samples from patients with tAMD, in addition to the 132 samples from patients with PCV. With this, the number of samples for nAMD totaled 212, and we confirmed that a power close to 0.9 was achieved.

### Cholesterol Efflux Capacity

The cholesterol efflux assay measures the capacity of serum acceptors to accept cholesterol released from the cells.^40^ Herein, the capacity of patient serum to mediate cholesterol efflux was measured using J774A.1 macrophages with a fluorometric cholesterol efflux assay kit (MAK192-1KT; Sigma-Aldrich). In brief, J774A.1 macrophages in log phase were suspended in Roswell Park Memorial Institute (RPMI) 1640 medium with 10% fetal bovine serum and seeded at a density of 1 × 10^5^ per well in 96-well plates. Cells were allowed to adhere by incubating the plates at 37° C with 5% CO_2_ for 2 hours. After that, cells were washed with RPMI 1640 medium without fetal bovine serum. Fluorescent-labelled cholesterol was then added to each well, and the plates were incubated again for 16 hours at 37° C with 5% CO_2_. Apolipoprotein B-containing lipoproteins (low-density lipoprotein [LDL] and very LDL [VLDL]) in the serum samples were depleted by adding polyethylene glycol to remove their background activities.^14^^,^^15^ After the incubation, cells were washed with RPMI 1640 without fetal bovine serum and treated with triplicate serum samples in RPMI medium for 4 hours at 37° C with 5% CO_2_. Then supernatants were transferred to a respective new 96-well plate and the fluorescence intensity was measured. Cell monolayers in the wells were lysed by the lysis buffer by incubating at room temperature (25° C) for 30 minutes. The cell lysate then was transferred to the respective wells of the new 96-well plate with the supernatant, followed by reading fluorescence intensity with excitement of 485 nm and emission of 523 nm. Cholesterol efflux capacity was measured as a percentage of fluorescent intensity of medium over fluorescent intensity of medium and cell lysate, as follows:

$$Cholesterol\;efflux\;capacity\;\left(\%\right)=\frac{Fluorescent\;intensity\;of\;medium}{Fluorescent\;intensity\;of\;medium\;and\;cell\;lysate}x100$$

### Lipid Subfractions

In addition to routine blood lipid measurements, lipid subfractions were measured by NMR-based metabolomics analysis in control, eAMD, and PCV samples (Nightingale, Ltd). The measurement produces the concentrations of three subclasses of VLDL, LDL, and HDL particles each. Lipoprotein subclasses were grouped as HDL 1 (7.3–7.7 nm), HDL 2 (7.8–8.2 nm), HDL 3 (8.2–8.8nm), HDL 4 (8.8–10 nm), and HDL 5 (10–13 nm),^41^ or extra-large, large, medium, and small based on size.

### Detailed Nuclear Magnetic Resonance Data Analysis

For the analysis on metabolites including lipid subfractions, fatty acids, and amino acids, all subfractions were log transformed and scaled to make comparable measurements, as suggested.^10^ In brief, associations of magnitudes were reported in units of standard deviation (SD) or odds ratio change per 1-SD increase in each metabolite. Bonferroni correction was applied to correct for multiple testing.

### Statistical Analysis

Statistical analysis was performed using R statistical software version 3.6.3 (R Foundation for Statistical Computing, Vienna, Austria). The analysis of variance and chi-square test were used to compare the baseline characteristics and routine lipoprotein profiles among the 4 groups. Multiple regression analysis was used to investigate the difference in the cholesterol efflux capacity among the 4 groups, adjusted for age, gender, and lipid-lowering drug. The Tukey test was used as a post hoc comparison. Associations between the cholesterol efflux capacity and the lipid subfractions also were examined with the Pearson correlation coefficient after distribution of normality was confirmed with the Shapiro-Wilk W test. Logistic regression was used to investigate risk factor of PCV among the lipoprotein profile. *P* values of less than 0.05 were considered significant.

## Results

### Characteristics of Patients

The baseline characteristics of participants are summarized in Table 1. In total, samples comprised 80 patients with eAMD, 212 patients with nAMD (80 with tAMD and 132 with PCV), and 110 age- and gender-matched control participants with a mean ± SD age of eAMD 67.1 ± 9.30 years, 68.7 ± 8.7 years (69.4 ± 7.9 years and 68.3 ± 9.2 years), and 66.7 ± 8.5 years, respectively. Age (*P* = 0.152), sex (*P* = 0.702), and use of lipid-lowering drugs (*P* = 0.319) were well balanced among these 4 groups.

**Table 1:** Baseline Characteristics of Study Groups

Variables	Control Group (n = 110)	Early Age-Related Macular Degeneration Group (n = 80)	Neovascular Age-Related Macular Degeneration Group (n = 212)		*P* Value∗†
			Typical Neovascular Age-Related Macular Degeneration Group (n = 80)	Polypoidal Choroidal Vasculopathy Group (n = 132)	
Age (yrs), mean ± SD	66.7 ± 8.5	67.1 ± 9.3	68.7 ± 8.7		0.1515
			69.4 ± 7.9	68.3 ± 9.2	
Male sex (%)	52.7	58.8	59.4		0.7015
			60.0	59.1	
Lipid-lowering drug use (%)	39.1	43.0	46.2		0.3188
			52.6	42.4	

SD = standard deviation.

∗ Analysis of variance used for age.

† Chi-square test used to for the proportion of men and the use of lipid-lowering drugs.

### Cholesterol Efflux Capacity

**Figure 1:**
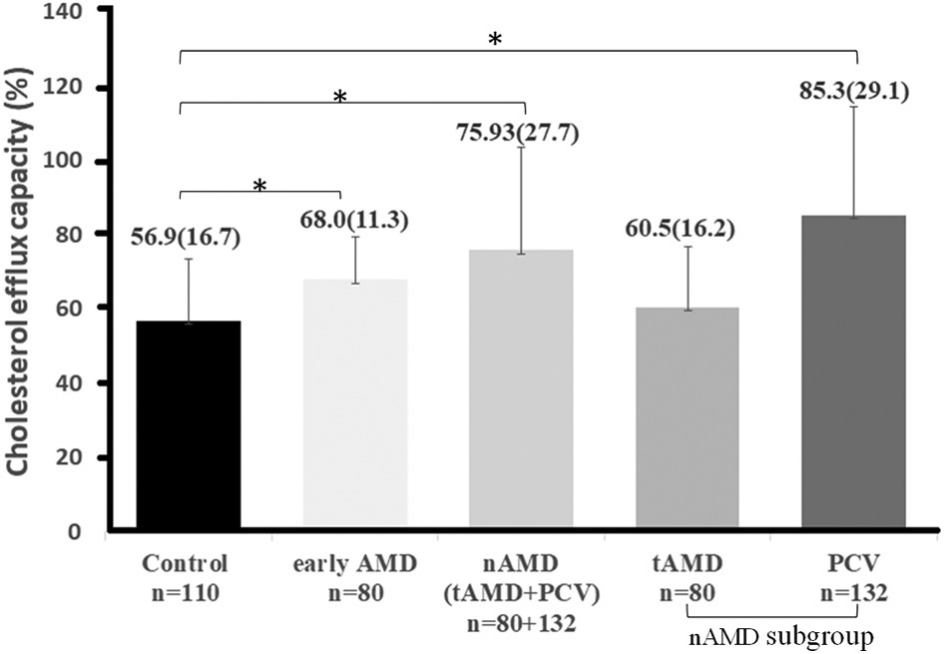
Bar graph showing cholesterol efflux capacity of control participants and patients with early age-related macular degeneration (AMD), neovascular AMD (nAMD), and polypoidal choroidal vasculopathy (PCV). A multiple linear regression model was used to compare cholesterol efflux capacity of the 3 groups, adjusting the various covariates, including factors affecting cholesterol efflux capacity such as age, gender, and lipid-lowering drug use. The generalized least squares estimation method was used in the multiple linear regression model for the heterogeneity of variance among the 3 groups. ∗*P* < 0.0083 was considered significant after Bonferroni correction. tAMD = typical neovascular age-related macular degeneration.

Serum from patients with eAMD and nAMD showed a significantly higher cholesterol efflux capacity than that of control participants after adjusting for age, sex, and use of lipid-lowering drugs (Fig 1). In a subgroup analysis of nAMD, cholesterol efflux was higher in patients with PCV, but not patients with tAMD, than in control participants.

### Lipoprotein Profiles

**Figure 2:**
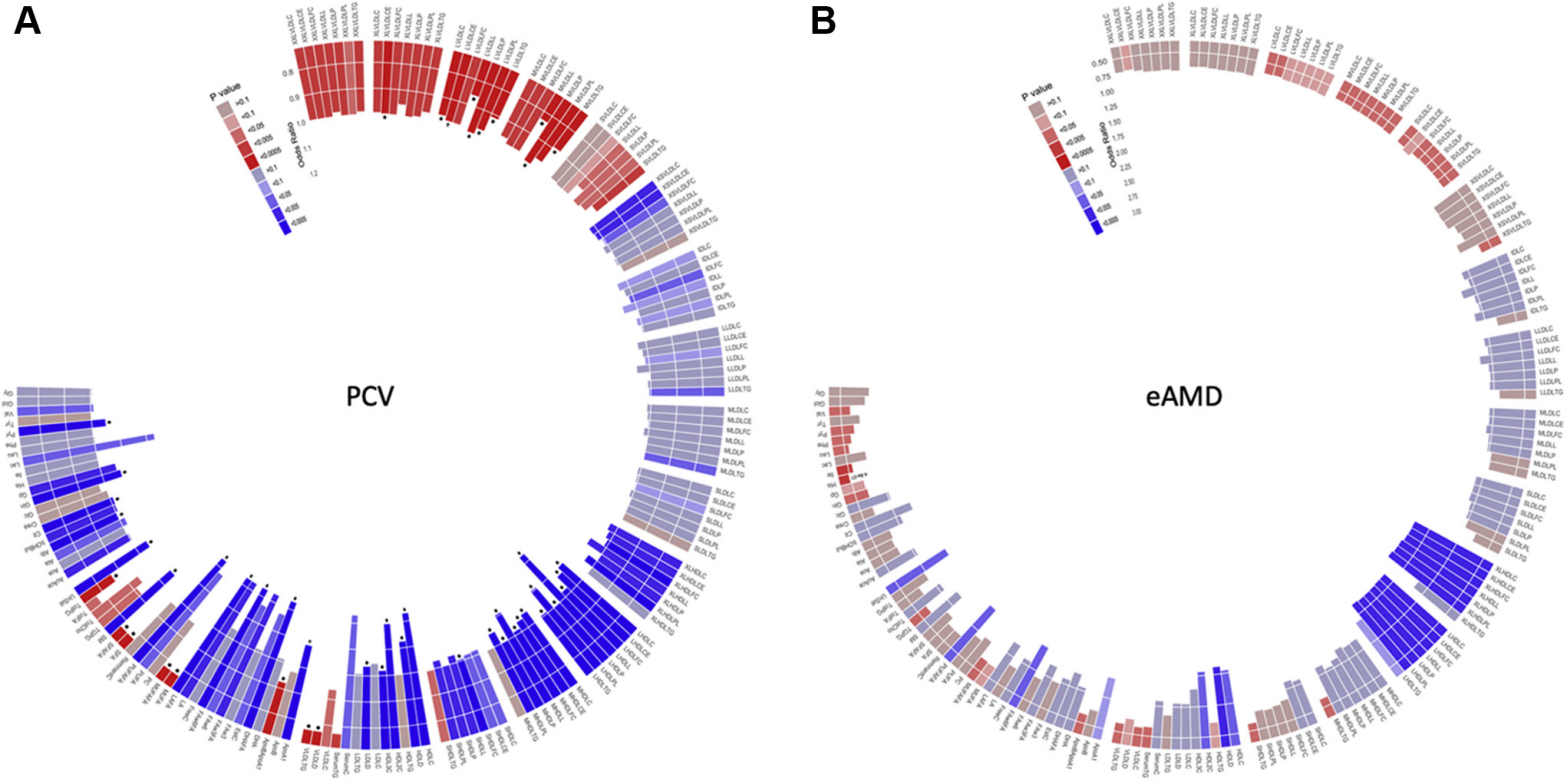
Graphs showing the association of metabolic variables in early age-related macular degeneration (eAMD) and polypoidal choroidal vasculopathy (PCV). Each bar represents the association with (A) PCV and (B) eAMD. The size of the bar is the odds ratio, and color refers to effect direction (blue is positive and red is negative) and significance. Dots indicate Bonferroni statistically significant metabolic variables corrected for age, sex, and lipid-lowering drug use (the statistically significance level was i < 0.00022 after Bonferroni correction). Labels describe the properties measured in each lipid subfraction. C = total cholesterol; CE = cholesterol ester; FC = free cholesterol; L = total lipid; P = concentration of particles; PL = phospholipid; TG = triglyceride.

A conventional clinical lipid assay showed that nAMD samples harbored higher levels of HDL and LDL cholesterol than those of control participants; the mean ± SD level of HDL cholesterol was 1.27 ± 0.34 mmol/1 and 1.46 ± 0.33 mmol/1 for control participants and patients with nAMD, respectively, and the mean ± SD level of LDL cholesterol was 3.17 ± 0.87 mmol/1 and 3.53 ± 1.03 mmol/1 for control participants and patients with nAMD, respectively (*P* < 0.02; Table 2). No differences were found in the levels of HDL and LDL cholesterol between patients with tAMD and those with PCV. In the eAMD samples, the levels of HDL and LDL were not significantly different from those of control participants, that is, the mean ± SD levels of HDL cholesterol and LDL cholesterol were 1.33 ± 0.45 mmol/1 and 3.08 ± 0.85 mmol/1 for eAMD (*P* > 0.05 for both). Lipoprotein subfraction analysis showed that the concentrations of very large and large HDLs and large, medium, and small VLDL or the ratio of apolipoprotein B to apolipoprotein A1 was high in eAMD samples, but not significantly different from those of control participants (Table 2; Fig 2 [abbreviations shown in Supplemental Table 1]). In PCV samples, large and medium HDLs and large and medium VLDLs as well as the ratio of apolipoprotein B to apolipoprotein A1 were significantly higher, whereas extra-large HDL and small VLDL levels were not different from those of control participants (Table 2; Fig 2). The mean diameter of HDLs and LDLs was larger and that of VLDLs was smaller in PCV samples compared with control samples (Table 2). The diameter of HDL was significantly larger in patients with eAMD versus control participants, whereas the diameter of LDL and VLDL in patients with eAMD was not different from that of control participants (Table 2).

**Table 2:** Comaparison of lipoprotein profiles

Lipoproteins	Unit	Mean±Standard Deviation				P Value						
		Control	Early Age-Related Macular Degeneration	Neovascular Age-Related Macular Degeneration		Overall	Control	Control	Control	Early Age-Related Macular Degeneration	Earlyl Age-Related Macular Degeneration	Typical Neovascular Age-Related Macular Degeneration
				Typical Neovascular Age-Related Macular Degeneration	Polypoidal Choroidal Vasculopathy							
Conventional clinical lipid assay												
HDL cholesterol	mmol/l	1.27 ± 0.34	1.33 ± 0.45	1.46 ± 0.33		**0.0002**	0.6918	**0.0045**	**0.0007**	0.1305	0.0731	0.9998
				1.46 ± 0.32	1.45 ± 0.34							
LDL cholesterol	mmol/l	3.17 ± 0.87	3.08 ± 0.85	3.53 ± 1.03		**0.0008**	0.9234	**0.0377**	**0.0388**	**0.0122**	**0.01**	0.9792
				3.57 ± 1.03	3.51 ± 1.03							
NMR spectroscopy												
Total particle(s) concentration												
ApoA1	mg/dl	1.43 ± 0.16	1.47 ± 0.18	—	1.55 ± 0.27	**0.0002**	0.49	—	**0.0001**	—	0.0537	—
HDL	10–6 μmol/l	8.13 ± 1.09	8.37 ± 1.06	—	9.07 ± 1.41	**< 0.0001**	**0.044**	—	**< 0.0001**	—	0.0727	—
LDL	10–7 nmol/l	4.38 ± 1.25	4.35 ± 1.20	—	4.44 ± 1.65	0.9671	-	—	-	—	-	—
VLDL and chylomicron	10–8 nmol/l	6.64 ± 2.98	5.76 ± 2.23	—	5.37 ± 2.25	**0.0075**	0.0816		**0.0081**	—	0.9512	—
Mean particle diameter, nm												
HDL		9.84 ± 0.24	9.96 ± 0.27	—	9.97 ± 0.23	**0.0001**	**0.0058**	—	**0.0002**	—	0.9631	—
LDL		23.54 ± 0.11	23.54 ± 0.10	—	23.59 ± 0.13	**0.0002**	0.8796	—	**0.0003**	—	**0.0147**	—
VLDL		37.85 ± 1.56	37.35 ± 1.54	—	37.05 ± 1.33	**0.0002**	0.0821	—	**< 0.0001**	—	0.3607	—
Lipoprotein subclass analysis												
HDL												
Very large	10–7 μmol/l	3.39 ± 1.69	4.51 ± 2.17	—	4.33 ± 1.92	**< 0.0001**	**0.0008**	—	**0.0005**	—	0.8166	—
Large	10–7 μmol/l	9.37 ± 4.33	11.63 ± 4.95	—	12.78 ± 4.96	**< 0.0001**	**0.0084**	—	**< 0.0001**	—	0.2587	—
Medium	10–7 μmol/l	19.48 ± 3.45	19.48 ± 3.27	—	22.25 ± 4.54	**< 0.0001**	1	—	**< 0.0001**	—	**< 0.0001**	—
Small	10–7 μmol/l	49.28 ± 4.97	47.95 ± 4.82	—	51.47 ± 6.82	**0.0002**	0.3203	—	**0.0109**	—	**0.0003**	—
IDL	10–8 nmol/l	9.58 ± 2.44	9.51 ± 2.43	—	9.92 ± 3.18	0.5258	—	—	—	—	—	—
LDL												
Large	10–8 nmol/l	15.86 ± 4.48	15.79 ± 4.28	—	16.27 ± 5.80	0.7573	—	—	—	—	—	—
Medium	10–8 nmol/l	12.87 ± 3.83	12.75 ± 3.67	—	12.98 ± 5.07	0.9422	—	—	—	—	—	—

HDL = high-density lipoprotein; IDL = intermediate density lipoprotein.; LDL = low-density lipoprotein; NMR = nuclear magnetic resonance; VLDL = very low-density lipoprotein; — = not available.

Data derived using an analysis of variance followed by the Tukey test. P < 0.05 was considered significant. Boldface indicates statistical significance.

### Association of Cholesterol Efflux Capacity and High-Density Lipoprotein Subfractions

**Figure 3:**
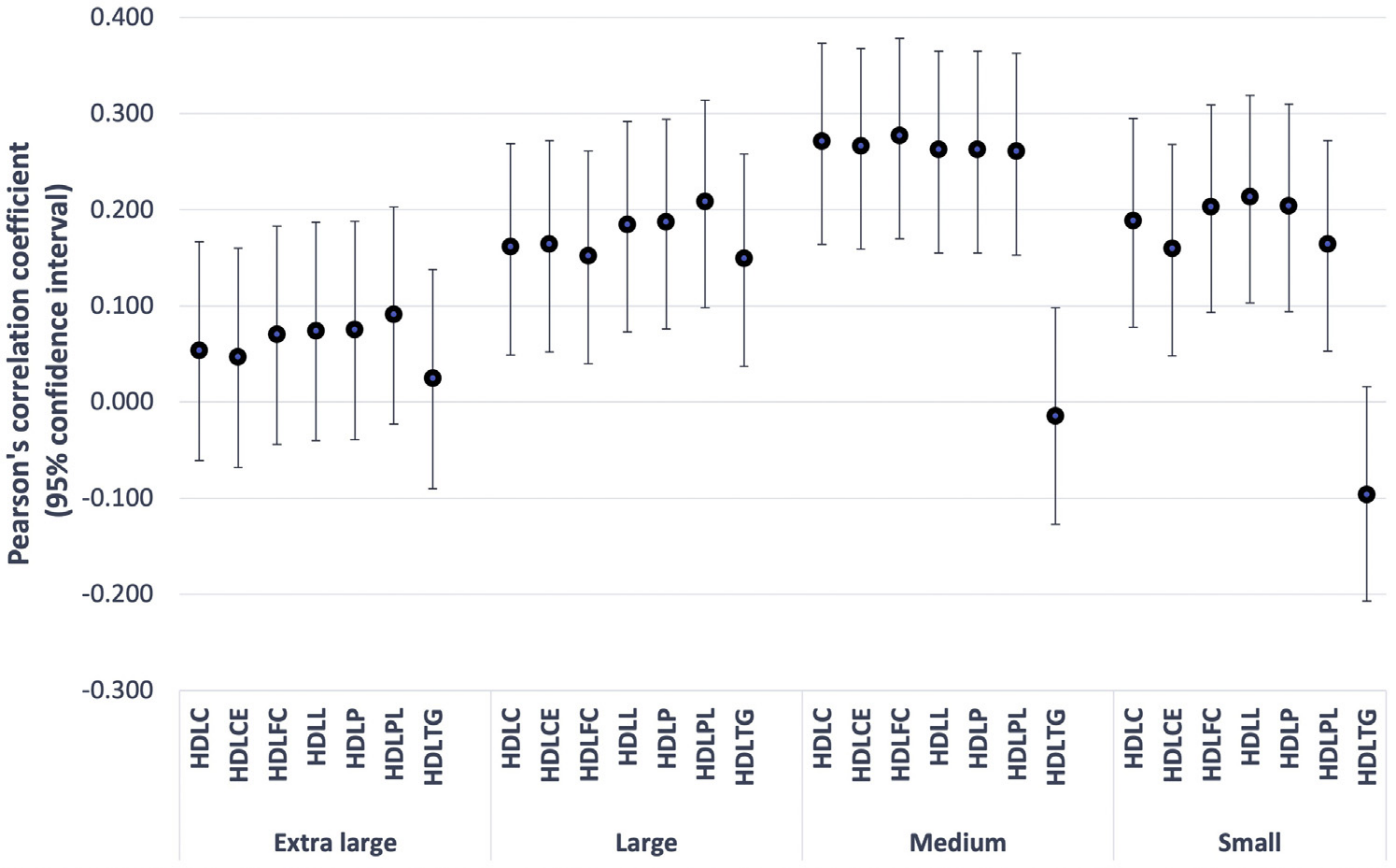
Graph showing Pearson’s correlation (with 95% confidence interval) between cholesterol efflux capacity and high-density lipoprotein (HDL) subfractions. The y-axis shows the coefficiency (*r*). Most of the small, medium, and large HDLs, but none of the 7 extra-large HDL fractions, were associated moderately with cholesterol efflux capacity. C = total cholesterol; CE = cholesterol ester; FC = free cholesterol; L = total lipid; P = concentration of particles; PL = phospholipid; TG = triglyceride.

Levels of HDL, HDL 2, HDL 3, and apolipoprotein A1 were associated moderately with cholesterol efflux capacity (*R* = 0.240, *R* = 0.235, *R* = 0.229, and *R* = 0.224, respectively; *P* < 0.001 for all), whereas LDL and VLDL cholesterols were not associated (*R* = 0.063 and *R* = –0.072, respectively; *P* > 0.05 for both). Among the 28 HDL subfractions, most of the small, medium, and large HDLs, but none of the 7 extra-large HDL fractions, were associated moderately with cholesterol efflux capacity (Fig 3).

## Discussion

In this study, we assessed serum cholesterol efflux capacity and subfractions of HDL particles analyzed by NMR spectroscopy. Serum cholesterol efflux capacity was the highest in participants with PCV, followed by those with eAMD. We demonstrated that serum cholesterol efflux capacity levels were not different between healthy control participants and patients with tAMD. In this study, small, medium, and large HDLs, but none of the 7 extra-large HDL fractions, were associated moderately with cholesterol efflux capacity. Higher rates of cholesterol efflux were associated modestly with increasing HDL composition, although it cannot completely account for the increased rates.^42^

### Cholesterol Efflux and Early Age-Related Macular Degeneration and Polypoidal Choroidal Vasculopathy

Five decades ago, it was first documented that drusen comprised at least 2 major components: mucopolysaccharide and lipid.^43^ Histologic studies performed in the 1990s demonstrated that the lipid-rich barrier in Bruch’s membrane is implicated as a cause of photoreceptor dysfunction and pigment epithelial detachment.^44^, ^45^, ^46^ It also was demonstrated that esterified or unesterified cholesterol in Bruch’s membrane increased with age in the macula, like arterial intima and other connective tissues, and the investigators assumed that age-related maculopathy and atherosclerotic cardiovascular disease may share common pathogenic mechanisms.^47^ However, systemic data from the current results, together with those from other clinical studies,^9^^,^^10^^,^^48^ remain controversial. A surprising finding in our study was that although the cholesterol efflux capacity was higher in participants with eAMD and PCV, it was not in those with tAMD. Probably the main element to be considered is that the lipid accumulation beneath the RPE is a life-long process, whereas the cholesterol efflux capacity merely reflects the functionality of HDL of the patients already affected by AMD. It is generally agreed that soft drusen form when secretions of functional RPE back up in the sub-RPE–basal lamina space by impaired egress across aged Bruch’s membrane–choriocapillary endothelium, and the mechanism contributing cholesteryl esters, the chemical form of cholesterol that is specific to drusen and Bruch’s membrane, starts in late adolescence. Additionally, RPE may be capable of managing unesterified cholesterol in various ways, including transfer to circulating HDL, complexing with endogenously synthesized apolipoproteins, conversion to an oxysterol capable of passing through cellular membranes, and release as microvesicles or exosomes.^28^ Several other possible explanations exist as follows.

First, enhanced cholesterol efflux capacity could be a compensatory mechanism to remove excess cholesterol accumulation in the RPE–Bruch’s membrane. In patients with AMD, a lipid wall, or densely packed lipoproteins in the sub-RPE space, may act as a barrier to block cholesterol removal from the choroid.^49^^,^^50^ In this regard, other mechanisms such as eliminating cholesterols, that is, metabolism to oxysterols by cytochrome P450 enzymes, or cholesterol uptake, that is, internalization of LDL through LDL receptors or scavenger, may have stronger impact on lipid accumulation, as have been demonstrated in animal studies.^51^ Second, it is also possible that the altered cholesterol efflux capacity is just an epiphenomenon to eAMD and PCV. The current study used macrophages to assess the serum cholesterol efflux capacity. Some differences exist between the formation of atherosclerotic plaque, that is, lipid deposition in the walls of systemic arteries in cardiovascular diseases, and esterified lipid-rich deposition and apolipoprotein B lipid deposition in the RPE–Bruch’s membrane in AMD. It is generally accepted that the increased capacity of the patient serum to accept cholesterol from cholesterol-laden macrophages represses atherosclerotic plaque formation; however, although macrophages mediate most cholesterol efflux to HDL, other pathways may be present.^52^ Less is known about the impact of HDL cholesterol efflux on eAMD or PCV. The cholesterol metabolism in the retina is a complex process, and the current results warrant further investigation using retinal cells such as RPE cells, neural retinal cells, and Müller cells.^53^

Our results showed that compared with patients with eAMD, systemic cholesterol efflux capacity was lower in patients with tAMD. This phenomenon is not easily explained, but a previous study also showed that cholesterol efflux capacity in patients with nAMD was not different from that in control participants.^48^ Cholesterol efflux capacity is reduced under acute inflammation, where the composition of HDL changes because of inflammation; such changes in HDL contribute to the reduction in cholesterol efflux capacity.^54^ A previous study demonstrated that patients with typical nAMD demonstrate increased C-reactive protein, interleukin 1b, and interleukin 6, whereas the levels of these cytokines in patients with PCV were not different from those in healthy control participants.^55^ Thus, tAMD, but not PCV, may be associated with low-grade systemic inflammation; however, further large-scale studies are needed for the validity of this conclusion. Additionally, higher HDL cholesterol levels were associated with an increased risk of any AMD, but risk estimates were significantly higher for eAMD than late AMD.^29^ These possible changes of HDL in nAMD, at least in part explain why cholesterol efflux capacity was lower in patients with tAMD compared with those with eAMD and PCV. Last but not least, although the retina is one of the few human organs whose cholesterol maintenance remains poorly understood, different pathways of cholesterol input, output, and regulation exist in the neuroretina and RPE region. For instance, in the photoreceptor outer segments where cholesterol content is the lowest of all retinal layers, cholesterol biosynthesis, catabolism, and regulation via liver X receptor and sterol element binding protein transcription factors are weak. Even in the RPE, where cholesterol content is higher, the gene expression also does not seem to be regulated by the sterol element binding proteins.^56^ Local cholesterol transport and efflux in the eye also may be subject to different and more complex regulation than systemic cholesterol efflux.

### Lipid Metabolites in Early Age-Related Macular Degeneration, Typical Neovascular Age-Related Macular Degeneration, and Polypoidal Choroidal Vasculopathy

Several studies investigated the association between the risk of AMD and lipid profile. The current study discovered significant associations of several lipid metabolites with PCV. Several epidemiologic studies demonstrated a significant association between high HDL cholesterol and an increased risk of soft drusen, nAMD, and geographic atrophy.^9^^,^^11^ The European Eye Epidemiology Consortium study^29^ clearly demonstrated that high HDL and low triglyceride levels were associated with an increased risk of AMD. Regarding the lipid fractions, the concentrations of extra-large HDL particles showed the most prominent association with AMD. The current study included both patients with AMD and those with PCV; the concentration of very large and large HDLs was numerically high in eAMD samples, and large and medium HDLs were significantly higher in PCV samples. This is in line with the previous study from our group showing that the increase in HDL particles in patients with nAMD was driven by an excess of medium particles.^57^ Thus, lipoprotein size associated with AMD or PCV in the current study was different from those reported by the European study.^29^ We assume that this is possibly the result of ethnic differences. Several other studies investigated lipid species in various ethnicities, albeit with different methods from the current study. A previous study of White patients with nAMD showed higher total serum lysophosphatidylcholine and serum lysophosphatidylcholine compared with those with geographic atrophy.^58^ In contrast, in an Asian population, platelet-activating factor was the key indicator of distinct lipid metabolism.^59^ In addition, 19 phosphatidylcholines, 8 sphingomyelins, 4 lysophosphatidic acids, 3 platelet-activating factors, 3 lysophosphatidylcholines, 2 sphingosines, 1 phytosphingosine, and 1 phosphatidylethanolamine were identified as discriminating metabolites.^59^ These studies suggest the importance of analyzing abundance profiling of lipid species in AMD across multiple ethnicities. Further studies using a standardized lipid subfraction analysis would be required to understand comprehensively the pathologic roles of systemic lipid in AMD.

### Study Limitations

This study has limitations. First, the study compared patients from a hospital-based cohort (the Asian AMD Phenotyping Study) with those from a population-based study (the SCES). Therefore, a potential may exist for selection bias. Second, although 40% to 50% of patients in each group were receiving lipid-lowering medications, we were not able to identify their specific types. Statins can be classified into hydrophilic or lipophilic, and taking very high-dose lipophilic statins may prevent progression to exudative AMD.^60^ Finally, we collected blood samples in nonfasting conditions. Because of the study design, we were not able to study all the participants in the fasting state. However, the European Eye Epidemiology Consortium study indicated that fasting and nonfasting sampling methods showed no interaction with the association between lipid levels and AMD in a sensitivity analysis,^10^ and we believe that lipid measurements in nonfasting samples remain relevant.

## Conclusions

In conclusion, we demonstrated that serum cholesterol efflux capacity was higher in patients with PCV and eAMD than in control participants, whereas that of tAMD was comparable with that of control participants. We also found that the lipid subfractions, that is, large and medium HDLs and large and medium VLDLs, were altered in PCV. In particular, our results indicate that small, medium, and large HDLs among all lipids play important roles in the pathogenesis of eAMD and PCV. The central theme of this study was to investigate the cholesterol efflux capacity of HDL, and this study serves as a first step toward understanding the diverse role of HDL. Further studies are needed to assess completely the diverse biological activities of HDL in AMD, including macular pigment transport, regulation of inflammation, and the local cholesterol transport system.
