# Supplementary material for: Serum Cholesterol Efflux Capacity in Age-Related Macular Degeneration and Polypoidal Choroidal Vasculopathy
Source: Ophthalmol Sci. 2022 Mar 16;2(2):100142. doi: 10.1016/j.xops.2022.100142 (PMC9562377; doi:10.1016/j.xops.2022.100142)
Supplement: Supplementary table [file mmc1.docx]

Abbreviations used for Figure2

| XXLVLDLP | Concentration of chylomicrons and extremely large VLDL particles |
| --- | --- |
| XXLVLDLL | Total lipids in chylomicrons and extremely large VLDL |
| XXLVLDLPL | Phospholipids in chylomicrons and extremely large VLDL |
| XXLVLDLC | Total cholesterol in chylomicrons and extremely large VLDL |
| XXLVLDLCE | Cholesterol esters in chylomicrons and extremely large VLDL |
| XXLVLDLFC | Free cholesterol in chylomicrons and extremely large VLDL |
| XXLVLDLTG | Triglycerides in chylomicrons and extremely large VLDL |
| XLVLDLP | Concentration of very large VLDL particles |
| XLVLDLL | Total lipids in very large VLDL |
| XLVLDLPL | Phospholipids in very large VLDL |
| XLVLDLC | Total cholesterol in very large VLDL |
| XLVLDLCE | Cholesterol esters in very large VLDL |
| XLVLDLFC | Free cholesterol in very large VLDL |
| XLVLDLTG | Triglycerides in very large VLDL |
| LVLDLP | Concentration of large VLDL particles |
| LVLDLL | Total lipids in large VLDL |
| LVLDLPL | Phospholipids in large VLDL |
| LVLDLC | Total cholesterol in large VLDL |
| LVLDLCE | Cholesterol esters in large VLDL |
| LVLDLFC | Free cholesterol in large VLDL |
| LVLDLTG | Triglycerides in large VLDL |
| MVLDLP | Concentration of medium VLDL particles |
| MVLDLL | Total lipids in medium VLDL |
| MVLDLPL | Phospholipids in medium VLDL |
| MVLDLC | Total cholesterol in medium VLDL |
| MVLDLCE | Cholesterol esters in medium VLDL |
| MVLDLFC | Free cholesterol in medium VLDL |
| MVLDLTG | Triglycerides in medium VLDL |
| SVLDLP | Concentration of small VLDL particles |
| SVLDLL | Total lipids in small VLDL |
| SVLDLPL | Phospholipids in small VLDL |
| SVLDLC | Total cholesterol in small VLDL |
| SVLDLCE | Cholesterol esters in small VLDL |
| SVLDLFC | Free cholesterol in small VLDL |
| SVLDLTG | Triglycerides in small VLDL |
| XSVLDLP | Concentration of very small VLDL particles |
| XSVLDLL | Total lipids in very small VLDL |
| XSVLDLPL | Phospholipids in very small VLDL |
| XSVLDLC | Total cholesterol in very small VLDL |
| XSVLDLCE | Cholesterol esters in very small VLDL |
| XSVLDLFC | Free cholesterol in very small VLDL |
| XSVLDLTG | Triglycerides in very small VLDL |
| IDLP | Concentration of IDL particles |
| IDLL | Total lipids in IDL |
| IDLPL | Phospholipids in IDL |
| IDLC | Total cholesterol in IDL |
| IDLCE | Cholesterol esters in IDL |
| IDLFC | Free cholesterol in IDL |
| IDLTG | Triglycerides in IDL |
| LLDLP | Concentration of large LDL particles |
| LLDLL | Total lipids in large LDL |
| LLDLPL | Phospholipids in large LDL |
| LLDLC | Total cholesterol in large LDL |
| LLDLCE | Cholesterol esters in large LDL |
| LLDLFC | Free cholesterol in large LDL |
| LLDLTG | Triglycerides in large LDL |
| MLDLP | Concentration of medium LDL particles |
| MLDLL | Total lipids in medium LDL |
| MLDLPL | Phospholipids in medium LDL |
| MLDLC | Total cholesterol in medium LDL |
| MLDLCE | Cholesterol esters in medium LDL |
| MLDLFC | Free cholesterol in medium LDL |
| MLDLTG | Triglycerides in medium LDL |
| SLDLP | Concentration of small LDL particles |
| SLDLL | Total lipids in small LDL |
| SLDLPL | Phospholipids in small LDL |
| SLDLC | Total cholesterol in small LDL |
| SLDLCE | Cholesterol esters in small LDL |
| SLDLFC | Free cholesterol in small LDL |
| SLDLTG | Triglycerides in small LDL |
| XLHDLP | Concentration of very large HDL particles |
| XLHDLL | Total lipids in very large HDL |
| XLHDLPL | Phospholipids in very large HDL |
| XLHDLC | Total cholesterol in very large HDL |
| XLHDLCE | Cholesterol esters in very large HDL |
| XLHDLFC | Free cholesterol in very large HDL |
| XLHDLTG | Triglycerides in very large HDL |
| LHDLP | Concentration of large HDL particles |
| LHDLL | Total lipids in large HDL |
| LHDLPL | Phospholipids in large HDL |
| LHDLC | Total cholesterol in large HDL |
| LHDLCE | Cholesterol esters in large HDL |
| LHDLFC | Free cholesterol in large HDL |
| LHDLTG | Triglycerides in large HDL |
| MHDLP | Concentration of medium HDL particles |
| MHDLL | Total lipids in medium HDL |
| MHDLPL | Phospholipids in medium HDL |
| MHDLC | Total cholesterol in medium HDL |
| MHDLCE | Cholesterol esters in medium HDL |
| MHDLFC | Free cholesterol in medium HDL |
| MHDLTG | Triglycerides in medium HDL |
| SHDLP | Concentration of small HDL particles |
| SHDLL | Total lipids in small HDL |
| SHDLPL | Phospholipids in small HDL |
| SHDLC | Total cholesterol in small HDL |
| SHDLCE | Cholesterol esters in small HDL |
| SHDLFC | Free cholesterol in small HDL |
| SHDLTG | Triglycerides in small HDL |
| VLDLD | Mean diameter for VLDL particles |
| LDLD | Mean diameter for LDL particles |
| HDLD | Mean diameter for HDL particles |
| SerumC | Serum total cholesterol |
| VLDLC | Total cholesterol in VLDL |
| RemnantC | Remnant cholesterol (non-HDL, non-LDL -cholesterol) |
| LDLC | Total cholesterol in LDL |
| HDLC | Total cholesterol in HDL |
| HDL2C | Total cholesterol in HDL2 |
| HDL3C | Total cholesterol in HDL3 |
| EstC | Esterified cholesterol |
| FreeC | Free cholesterol |
| SerumTG | Serum total triglycerides |
| VLDLTG | Triglycerides in VLDL |
| LDLTG | Triglycerides in LDL |
| HDLTG | Triglycerides in HDL |
| TotPG | Total phosphoglycerides |
| TGPG | Ratio of triglycerides to phosphoglycerides |
| PC | Phosphatidylcholine and other cholines |
| SM | Sphingomyelins |
| TotCho | Total cholines |
| ApoB | Apolipoprotein B |
| ApoBApoA1 | Ratio of apolipoprotein B to apolipoprotein A-I |
| TotFA | Total fatty acids |
| UnSat | Estimated degree of unsaturation |
| DHA | 22:6, docosahexaenoic acid |
| LA | 18:2, linoleic acid |
| FAw3 | Omega-3 fatty acids |
| FAw6 | Omega-6 fatty acids |
| PUFA | Polyunsaturated fatty acids |
| MUFA | Monounsaturated fatty acids; 16:1, 18:1 |
| SFA | Saturated fatty acids |
| DHAFA | Ratio of 22:6 docosahexaenoic acid to total fatty acids |
| LAFA | Ratio of 18:2 linoleic acid to total fatty acids |
| FAw3FA | Ratio of omega-3 fatty acids to total fatty acids |
| FAw6FA | Ratio of omega-6 fatty acids to total fatty acids |
| PUFAFA | Ratio of polyunsaturated fatty acids to total fatty acids |
| MUFAFA | Ratio of monounsaturated fatty acids to total fatty acids |
| SFAFA | Ratio of saturated fatty acids to total fatty acids |
| Glc | Glucose |
| Lac | Lactate |
| Pyr | Pyruvate |
| Cit | Citrate |
| Glol | Glycerol |
| Ala | Alanine |
| Gln | Glutamine |
| Gly | Glycine |
| His | Histidine |
| Ile | Isoleucine |
| Leu | Leucine |
| Val | Valine |
| Phe | Phenylalanine |
| Tyr | Tyrosine |
| Ace | Acetate |
| AcAce | Acetoacetate |
| bOHBut | 3-hydroxybutyrate |
| Crea | Creatinine |
| Alb | Albumin |
